# Spinal Epidural Abscess: A Review with Special Emphasis on Earlier Diagnosis

**DOI:** 10.1155/2016/1614328

**Published:** 2016-12-01

**Authors:** Allison Bond, Farrin A. Manian

**Affiliations:** Department of Medicine, Massachusetts General Hospital, Harvard Medical School, Boston, MA, USA

## Abstract

Spinal epidural abscess (SEA) is an uncommon but serious condition with significant morbidity and mortality. The prognosis of SEA is highly dependent on the timeliness of its diagnosis before neurological deficits develop. Unfortunately, often due to its nonspecific presentation, such as back pain, the diagnosis of SEA may be delayed in up to 75% of cases. Although many risk factors for SEA can be found in the published literature, their utility is limited by their frequent lack of objective evidence, numerousness, and absence in a significant proportion of cases. In this review, we call for a more discriminate evidence-based use of the term “risk factor” when discussing SEA and explore several approaches to its earlier diagnosis, including a simple algorithm based on its pathophysiology and serum C-reactive protein or erythrocyte sedimentation rate.

## 1. Introduction

Since its original postmortem description more than 250 years ago by Giovanni Morgagni, spinal epidural abscess (SEA) has often evaded timely diagnosis, with up to 75% of cases misdiagnosed on their initial healthcare encounter [1, 2]. Such delays in diagnosis—and therefore of timely therapy—may lead to significant morbidity and mortality [3]. Because the signs and symptoms of SEA are often nonspecific, a high index of suspicion is key to making a timely diagnosis. The goal of this article is to provide clinicians with a general overview of SEA with a special emphasis on critical examination of its reported risk factors and exploration of potential approaches to its earlier diagnosis.

## 2. Epidemiology

### 2.1. Incidence

Although SEA is uncommon, its incidence is rising. From 1975 to 1998, for example, the incidence of SEA rose from 0.2–1.2 to 2.5–3.0 cases per 10,000 hospital admissions [3]. This number is expected to rise further given a likely increase in the prevalence of patients at risk of SEA [3, 4]. Heightened awareness of SEA and increased use of sensitive imaging techniques such as magnetic resonance imaging (MRI) may also contribute to the rise in the number of reported cases [5, 6]. Although male predominance with higher prevalence between the fifth and seventh decade of life is often reported in SEA, a wide age distribution affecting virtually all age groups is also commonly described [7].

### 2.2. Risk Factors

Published risk factors for SEA are numerous and include diabetes mellitus (DM), intravenous drug use (IVDU), alcohol abuse, infection with human immunodeficiency virus (HIV), degenerative joint disease, recent trauma or surgery, and the presence of spinal stimulators or catheters [3, 7–9]. Local or systemic infections are also commonly listed as risk factors; these include skin and soft tissue infections, osteomyelitis, urinary tract infection (UTI), sepsis, and indwelling vascular access infection. In addition, hypertension, chronic obstructive pulmonary disease (COPD), chronic liver or kidney disease, nerve acupuncture, tattooing, epidural analgesia, and nerve block are also thought to increase the likelihood of SEA [8, 9]. Although reported risk factors are often intended to heighten awareness and facilitate earlier diagnosis of SEA, their routine clinical application has several limitations.

First, published risk factors or predisposing conditions for SEA are often derived from case reports, small case series, and literature reviews, which often fail to distinguish preexisting or potentially causative factors from coexisting conditions that may have very little role in the causation of SEA [3–15]. This is an important distinction because a risk factor must not only be shown to precede the disease but also be independently associated with its development [16–18]. For example, a frequently cited “meta-analysis” article implausibly lists several conditions such as “hepatitis,” “vaginal infection,” and “typhus” as risk factors or sources of infection for SEA based on descriptive case reports [7]; parenthetically, despite its title, no meta-analysis was performed [19]. Another article lists hypertension as a predisposing factor for SEA by “diminishing effective immune responses,” without offering any further explanation on its mechanism [9]. Even when more plausible conditions such as degenerative spinal disease, psoas abscess, or spinal trauma are listed as risk factors, their mere presence as a coexisting condition or a complication of the SEA itself is difficult to exclude [7]. For example, with more than 80% of adults in their 50s or older having lumbar spondylosis, a high prevalence of degenerative spinal disease in older patients with SEA would not be surprising [20] and cannot automatically be assumed to be related to this condition. Similarly, psoas abscess may not only precede SEA but also complicate it [19, 21]. Even spinal trauma cannot be assumed to uniformly have a causative role in SEA given the potential for recall bias in its self-reporting [22].

Another limitation of the current risk factors is that formal studies to elucidate their independent association with SEA through statistical analyses have often involved a relatively small number of patients with contradictory results. For example, in a case-control study of patients admitted to a rehabilitation facility following SEA or spinal trauma, those with SEA were more likely to have used IV drugs but were not more likely to have DM or a history of alcohol abuse [23]. Another study involving patients with SEA or spinal subdural abscess found that a history of DM, IVDU, spinal trauma, and degenerative spinal disease were not associated with SEA, while obesity and alcoholism were more predictive [24].

The sheer number of published risk factors also limits their clinical utility in the diagnosis of SEA. Some papers report as many as 50 “risk factors and sources of infection,” which often reflect common comorbidities or conditions often associated with* S. aureus* bacteremia, including DM, hemodialysis, HIV infection, heart disease, cancer, alcohol abuse, IVDU, COPD, and soft tissue infections [7, 25, 26]. Further diminishing the utility of risk factors in considering a diagnosis of SEA is their apparent absence in up to 20% to 50% of cases [5, 27–29].

## 3. Pathophysiology

### 3.1. Sources of Infection

SEA develops when microorganisms gain access to the epidural space via hematogenous spread from a distant source such as skin or respiratory or urinary tract; from contiguous foci of infection such as the psoas muscle or vertebra; or by direct inoculation through spinal instrumentation, injection, or catheter placement [3]. Of these mechanisms, hematogenous spread is the most common, accounting for about half of all cases [8], followed by direct spread from a contiguous focus of infection (about one-third); no source is identified in the remaining cases [8]. Interestingly, the location of SEA among patients with IVDU history may correlate with the site of drug injection, with cervical and lumbar spine more likely to be associated with upper and lower extremity sites of injection, respectively [2].

Another potential route of hematogenous infection is the pelvic cavity's venous drainage system, which connects with those of the spine and cranium via the spinal veins and forms Batson's plexus [10]. This valveless venous network may facilitate spread of organisms from pelvic organs (such as the urinary bladder) to the spinal column. More distant sources of infection, such as the oral cavity, should also be considered [30–32], particularly when the source of SEA is not readily apparent.

### 3.2. Mechanism of Neurological Deficits

Although neurological deficits caused by SEA are often attributed to its direct compression on the spinal cord resulting in ischemia and injury [9], local circulatory disruption due to venous stasis or thrombosis of spinal arteries has also been implicated [9, 12, 33]. This hypothesis may explain the difficulty in predicting the tempo of neurological complications following symptom onset in SEA, a view that is not universally endorsed, however [3, 22, 34].

## 4. Pathogens

Although SEA may be caused by a countless number of organisms,* Staphylococcus aureus* accounts for the majority of cases (60%–90%) with methicillin-resistant* S. aureus* (MRSA) accounting for an increasing number [2, 3, 8, 35–37]. Among aerobic Gram-negative bacilli,* Escherichia coli* often causes SEA in patients with UTI, while* Pseudomonas aeruginosa* may be the culprit in the setting of IVDU. Other pathogens such as mycobacteria, including* Mycobacterium tuberculosis*, tend to target immunosuppressed patients, while staphylococcal species other than* S. aureus* and fungi such as* Candida *species are often associated with spinal instrumentation or injection. SEA caused by an environmental fungus,* Exserohilum rostratum*, was recently reported in a multistate outbreak involving contaminated corticosteroid injections [38].

## 5. Diagnosis

### 5.1. Symptoms and Signs

Back pain is the most common presenting symptom of SEA, occurring in 70% to 100% of patients [8, 15, 27, 39]. The pain tends to be severe and localized with a duration of 1 day to 2 months prior to presentation [3, 8, 38]. Fever is found in about 50%, and back tenderness has been reported in 17% to 98% of cases [3, 8, 39–41]. Neurological manifestations, such as motor weakness, radiculopathy, and bladder and bowel dysfunction, have been reported in up to half of the cases [3]. Atypical manifestations of SEA, such as sudden paralysis, abdominal pain, headache, and bowel dysfunction, have also been reported [42–44]. It should be emphasized that the classic triad of back pain, fever, and neurological deficits is found in only a minority of patients with SEA [8].

### 5.2. Laboratory Abnormalities

Leukocytosis has been reported in 60% to 80% of patients presenting with SEA [3, 15]. Serum C-reactive protein (CRP) and erythrocyte sedimentation rate (ESR) have higher sensitivities and are almost uniformly elevated in patients with SEA [3]. Although blood cultures yield an organism in only about half of patients with SEA, they should be obtained routinely as they may guide antibiotic selection when the tissue cultures are not helpful or available [33].

### 5.3. Radiographic Abnormalities

Gadolinium-enhanced MRI is the radiographic test of choice for the detection of SEA with greater than 90% sensitivity and specificity [2, 3]. Some investigators have advocated imaging of the entire spine to exclude noncontiguous SEA in patients with symptom duration of at least one week prior to presentation, the presence of concomitant area of infection outside the spinal region, and when ESR is greater than 95 mm/h [45]. In patients with persistent symptoms but an initially unremarkable MRI, repeat testing in 2 to 3 weeks should be considered [3, 38]. CT myelography is usually not recommended because of its invasive nature and the potential for inadvertent contamination of the subarachnoid space [3]. Computed tomography (CT) scan with intravenous contrast has lower sensitivity, particularly in the early stages of SEA, and should be considered only when MRI cannot be performed [3, 8]. Nuclear medicine studies, such as technetium and indium isotope scans, have very little role in the diagnosis of SEA due to their suboptimal sensitivity and poor anatomical resolution [10, 15].

### 5.4. Invasive Diagnostic Tests

Once SEA is suspected radiographically, direct sampling of the infected fluid or tissue via image-guided biopsy should be performed to help confirm the diagnosis and direct antimicrobial therapy [3]. In some cases, the need for diagnostic aspiration or sampling may be obviated by isolation of a common etiologic pathogen, such as* S. aureus*, from blood cultures. Lumbar puncture should be avoided in patients with suspected SEA due to the risk of spread of infection into the subarachnoid space [3].

## 6. Treatment

Antibiotics should be administered as soon as cultures from blood and other possible sources of infection have been obtained [1]. Antimicrobial therapy should not be withheld preoperatively in patients with suspected sepsis or neurological symptoms, given the high degree of concordance between blood and abscess cultures and the importance of timely therapy [3, 46]. Routine empiric coverage of staphylococci (including MRSA), streptococci, and aerobic Gram-negative pathogens such as a combination of vancomycin with either piperacillin-tazobactam or a third- or fourth-generation cephalosporin (such as ceftazidime or cefepime) is often recommended [3, 8, 9]. Once the causative pathogen has been isolated, deescalation of therapy is advised. Patients at risk of fungal SEA should also receive an antifungal agent such as voriconazole or amphotericin B [38].

In addition to antimicrobial therapy, prompt surgery is indicated in most cases of SEA [3, 8, 27, 40, 46, 47]. Successful treatment of SEA with medical therapy alone in selected patients has also been reported [27, 40, 46, 48–51]. However, caution is advised when interpreting these reports because of their observational nature and the potential for selection of less severely ill patients for medical therapy alone [3]. A recent review article concluded that most studies advocate for early (within 24 hours of diagnosis) surgery due to high failure rates and a significant risk of morbidity (22% for permanent paralysis) and mortality (3% to 25%) with nonoperative treatment alone [47]. In contrast, medical therapy alone may be favored in patients with panspinal SEA involvement or those with complete paresis for 72 hours or more or when surgery is deemed too risky [3, 15, 40, 46].

Recommendations for the duration of antibiotic therapy vary widely from 4 weeks to 16 weeks depending on many factors, including comorbidities and concurrent presence of vertebral osteomyelitis [3]. Although the optimal duration of parenteral antibiotics is not always defined, most patients receive at least 2 to 4 weeks of therapy when vertebral osteomyelitis is not suspected [3]. Patients with* M. tuberculosis*-associated SEA should receive 6 months to 1 year of appropriate therapy (e.g., isoniazid and rifampin). Whenever possible, infected indwelling spinal hardware (such as a spinal stimulator or catheter) should be removed. Close monitoring for signs and symptoms of relapse after completion of antimicrobial therapy is essential in the management of SEA. Of note, one study found a treatment failure rate of 28% in patients with SEA caused by MRSA [52].

## 7. Prognosis

The outcome of SEA is often assessed based on mortality and recovery from neurological deficits [3]. Although the mortality rate has fallen significantly from 80% in the preantibiotic era to 2% to 20% in recent decades, many patients continue to die from this condition [3, 8, 53]. Death is generally due to overwhelming sepsis and typically occurs in patients with multiple comorbidities [3].

About one-third of survivors may have a poor neurological outcome often due to diagnostic delays [3]. In fact, a patient's ultimate neurological outcome correlates strongly with the severity and duration of neurological deficits prior to surgery [3]. The presence of spinal cord-related deficits upon presentation may also be a risk factor for failure of medical therapy [54]. These findings underscore the importance of diagnosis of SEA before neurological complications develop.

## 8. Potential Strategies for Earlier Diagnosis

A major obstacle to the timely diagnosis of SEA is the nonspecificity of its signs and symptoms. In addition, as previously discussed, for the clinician evaluating a patient with severe back pain, the utility of published risk factors is limited due to their uncertain role in SEA causation, seemingly countless number, and absence in a significant proportion of patients who are ultimately diagnosed with SEA.

Only a few studies have examined specific strategies for reducing delays in the diagnosis of SEA. A single-center study of routine MRI screening in emergency room patients suspected of having SEA yielded a diagnosis of SEA in less than 7% of patients [41] despite more than half of the patients having a history of IVDU. This study questions the cost-effectiveness of routine MRI for back pain, even among patients considered at high risk for SEA. Of note, none of the patient demographics studied in this study, including history of IVDU, was helpful in distinguishing patients who had SEA from those who did not.

Another single-center study evaluated the impact of a decision guide on diagnostic delays of SEA in patients presenting to an ED with back pain [1]. One or more risk factors for SEA were present in 100% of patients ultimately diagnosed with SEA, as compared to 23% of controls. In addition, an elevated ESR or CRP was found in 100% and 87% of SEA cases, respectively. A decision-making guide evaluating patients with progressive neurological deficits or elevation of either the ESR or the CRP—combined with the presence of one or more prespecified risk factors for SEA, fever, or radicular pain—was suggested. For all other patients without an obvious source of pain, discharge with follow-up was recommended. Although a decrease in diagnostic delays was observed following adoption of the algorithm compared to historical data, the presence of a risk factor in 100% of cases (compared to frequently lower rates in other studies), the reported inconsistent application of the algorithm by providers, and lack of an active surveillance system for follow-up of patients with risk factors but no ESR and CRP on file made interpretation of the data problematic [1].

Another potential strategy for early detection of SEA consists of obtaining MRI in all patients with severe back pain. However, the feasibility and cost-effectiveness of this approach may be questioned in the current era of stewardship of healthcare resources [41].

**Figure 1:**
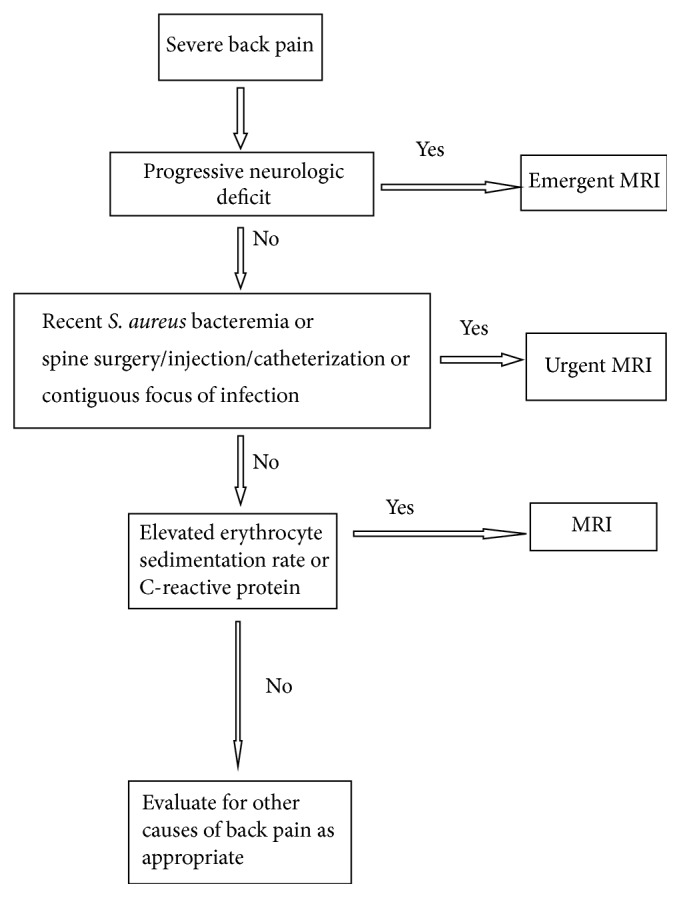
The proposed algorithm for SEA diagnosis in patients presenting with severe back pain.

We propose an alternative strategy in the evaluation of patients with severe back pain which uses a simple algorithm (Figure 1). This algorithm is based on the pathophysiology of SEA, its often unpredictable clinical course, the high sensitivities of ESR and CRP in this condition, and a limited number of risk factors for which a direct role in the causation of SEA can be easily invoked (i.e.,* S. aureus* bacteremia, contiguous focus of infection, and spinal injection/instrumentation). Accordingly, we recommend that patients with severe back pain and progressive neurological deficits undergo emergent MRI, or a CT if MRI is contraindicated. In the absence of progressive neurological deficits, those with recent* S. aureus* bacteremia or spinal injection/instrumentation should undergo urgent (within 24 h) MRI. In all other patients, ESR and CRP should be obtained, and if either is elevated, MRI should be considered, with its timing contingent upon the overall assessment of the patient, severity of pain, and the likelihood of noninfectious explanations for the back pain. If both ESR and CRP are normal, SEA would be much less likely and further workup for noninfectious causes may be pursued as appropriate.

We further suggest that, in patients without back pain but at high risk of SEA because of a recent bacteremic illness (particularly caused by* S. aureus*), routine examination for spinal tenderness should be considered. This strategy is not dissimilar to searching for signs of endocarditis, such as new cardiac murmurs or embolic lesions, in patients with* S. aureus* bacteremia [55]. Of note, in a recent study of patients with MRSA bacteremia, the rate of SEA was the same as that of endocarditis (4% each) [56].

## 9. Conclusion

SEA is an uncommon but potentially devastating condition that continues to challenge the diagnostic skills of many clinicians. Reliance on published risk factors to help reduce diagnostic delays in SEA is limited by their seemingly countless number and their absence in a significant proportion of patients. More practical and feasible approaches to earlier diagnosis of SEA are sorely needed.
